# The effect of paracetamol on care dependency and daily functioning in persons with advanced dementia living in long-term care facilities

**DOI:** 10.1186/s12877-024-04795-8

**Published:** 2024-03-22

**Authors:** Paulien H van Dam, Wilco P Achterberg, Bettina S Husebo, Monique AA Caljouw

**Affiliations:** 1 University Network for the Care sector Zuid-Holland, Leiden University Medical Center, Leiden, The Netherlands; 2 Department of Public Health and Primary Care, Leiden University Medical Center, P.O. Box 9600, Leiden, 2300 RC The Netherlands; 3 Department of Global Public Health and Primary Care, Centre for Elderly – and Nursing Home Medicine, University of Bergen, Bergen, Norway; 4 Municipality of Bergen, Bergen, Norway

**Keywords:** Quality of life, Dementia, Paracetamol, Acetaminophen, Nursing home, Care dependency, Daily functioning

## Abstract

**Background:**

Pain medication may have an impact on the quality of life (QoL) in persons with dementia, but may also influence care dependency and daily functioning. The aim of this study is to investigate the effect of regularly scheduled paracetamol on care dependency and daily functioning in persons with advanced dementia with low QoL living in long-term care facilities.

**Methods:**

The Quality of life and Paracetamol In advanced Dementia (Q-PID) study was a (block) randomized double-blind placebo-controlled crossover trial with paracetamol and placebo across seventeen long-term care facilities across 9 care organizations in the western region of the Netherlands. Participants were ≥ 65 years, had advanced dementia (Global Deterioration Scale 5–7), and low QoL (QUALIDEM-6D score ≤ 70). Measurements were performed by nursing staff at the start and at the end of each treatment period of six weeks. Repeated linear mixed models were used to compute differences between randomization groups, with adjustment for period and order effects, and psychotropic use.

**Results:**

Ninety-five persons (mean age of 83.9 years, 57.4% female) were enrolled in the Q-PID study. The mean Care Dependency Scale total score was 37.8 (Standard Deviation [SD] 12.9) and the mean Katz-15 total score was 11.9 (SD 2.4). Repeated linear mixed models showed no difference in mean differences of care dependency (paracetamol − 1.0 [95% Confidence Interval (CI) -2.4-0.3], placebo + 0.1 [-1.3-1.5]), and daily functioning (paracetamol + 0.2 [95% CI -0.2-0.6], placebo + 0.1 [-0.3-0.4]).

**Conclusions:**

Compared to placebo, no effect of scheduled administration of paracetamol was found on care dependency and daily functioning in persons with advanced dementia with low QoL. Future research should focus on which specific items of care dependency need special attention to improve the care for persons with advanced dementia. A multi-domain approach is needed to enhance and/or maintain QoL of persons with advanced dementia.

**Trial registration:**

Netherlands Trial Register (NTR6766); http://www.trialregister.nl/trialreg/admin/rctview.asp?TC=6766; Trial registration date: 20/10/2017.

## Background

Pain has a considerable impact on care dependency and daily functioning in older persons living in Long-term care facilities (LTCF) [1–3], and may also lead to a lower quality of life (QoL) [4, 5]. 

Several studies found a positive effect of paracetamol in persons with advanced dementia, e.g., improving social interaction and engagement in activities [6], and daily functioning [7]. 

As pain medication may diminish pain [7] it may be hypothesized that it could reduce the negative impact of pain on care dependency and daily functioning of persons with advanced dementia. Furthermore, paracetamol may have a positive impact on wellbeing, but the working mechanism of paracetamol has not been completely clarified so far [8]. The Quality of life and Paracetamol In advanced Dementia (Q-PID) study assessed the effect of regularly scheduled administration of paracetamol (acetaminophen) on QoL, discomfort, pain, neuropsychiatric symptoms, care dependency, and daily functioning of persons with advanced dementia with low QoL living in LTCF. Recently, the first results of the Q-PID study, i.e., the effect of paracetamol (acetaminophen) on QoL, discomfort, pain and neuropsychiatric symptoms, were published [9]. No treatment effect in favour of paracetamol was found on these four outcomes. The aim of this study is to investigate the effect of regularly scheduled paracetamol on both care dependency and daily functioning in persons with advanced dementia with low QoL living in LTCF.

## Methods

The present study was conducted within the framework of the Q-PID study, and investigated two secondary outcome measures of this study, i.e., care dependency and daily functioning. The Q-PID study was a (block) randomized double-blind placebo-controlled crossover trial, performed between January 2018 and June 2019 in 17 LTCF across 9 care organizations in the western region of the Netherlands that are members of the University Network for the Care sector South Holland (UNC-ZH). Persons aged ≥ 65 years with advanced dementia (Global Deterioration Scale [GDS [10]] score of 5–7) and low QoL (QUALIDEM-6D total score ≤ 70) were included in this study. The QUALIDEM-6D as instrument to measure QoL in persons with advanced dementia was chosen because it has the widest set of measurement properties reported with satisfactory content and construct validity, and test-retest and inter-observer reliability [11, 12]. It was therefore the recommended observational instrument for assessing QoL in LTCF residents with dementia in a study by Aspden et al. [13].

Participants did not use pain medication daily and pain was not assessed prior to the study, assuring that participants were enrolled having low QoL. Each participant was randomized into one of the two randomization groups: one group received paracetamol for 6 weeks (3 g per day for the first 4 weeks, 2,5 gram per day in week 5 and 6), followed by a ‘wash-out’ week without study medication, and 6 weeks placebo. The second group received placebo for 6 weeks, followed by a ‘wash-out’ week without study medication, and 6 weeks paracetamol. Demographic characteristics were collected by the elderly care physician and nursing staff at baseline. Measurements were performed by nursing staff at the start and right before the end of each treatment period (at baseline, and before the end of weeks 6, 7, and 13). Detailed information about the study design, the intervention, and the results on the outcomes of QoL, discomfort, pain and neuropsychiatric symptoms can be found elsewhere [9, 14]. 

### Outcome measures

#### Care dependency

The Care Dependency Scale (CDS), which consists of components of care, was used to measure care dependency [15–17]. For each of the 15 items of the CDS, the nursing staff assessed the extent to which the person with dementia was able to perform a care task without assistance on a scale of 1 (completely dependent) to 5 (completely independent). The total score of the CDS ranges from 15 (completely care dependent) to 75 (completely care independent).

#### Daily functioning

(Instrumental) Activities of Daily Living (ADL) were measured with the Katz-15 scale, which comprises the six ADL items of the Katz-6 questionnaire (do you need help with bathing, dressing, using toilet, transfer to and from a chair, incontinence, and ability to eat without help?) [18], seven items of the Lawton Instrumental Activities of Daily Living (LIADL; do you need help with using a telephone, shopping, preparing food, performing household tasks, travelling, taking medication and handling own finances?) [19], and two extra questions whether the person needs help with combing hair/shaving, and walking about [20]. The Katz-15 scale ranges from 0 (no help needed with (i)ADL tasks) to 15 (help needed in all 15 items of (i)ADL).

#### Pain

In the Q-PID study, pain was monitored at baseline, and after 6, 7, and 13 weeks using the Mobilization-Observation-Behaviour-Intensity-Dementia Pain Scale 2 (MOBID-2) [21–23]. With this pain scale, nursing staff measured musculoskeletal pain during morning care, when guiding the person with dementia with movement of arms and legs, lying on both sides of the body, and sitting up on the bed (Part 1). MOBID-2 Part 2 consists of five items and assesses pain coming from head, skin, and internal organs. The nursing staff scored the pain of the person with dementia on a scale of 0 (no pain) to 10 (worst pain possible), based on the facial expression, vocalization, and defending behaviour during the movements, and based on the assessment of the five items in part 2. A score of ≥ 3 is regarded as clinically relevant pain [21]. 

### Statistical analysis

Differences between randomization groups were computed using unpaired t-tests for normally distributed numerical data, one-way ANOVA tests for non-normally distributed data, and χ^2^ tests for categorical data. A *p* value < 0.05 will be considered statistically significant. Since order and period effects were found for the main outcome QoL, repeated linear mixed models were used, with adjustment for these effects plus the use of psychotropics, as the number of psychotropic users was statistically different at baseline in the randomization groups. For details on sample size calculation, see the protocol article of the Q-PID study [14]. 

## Results

**Fig. 1:**
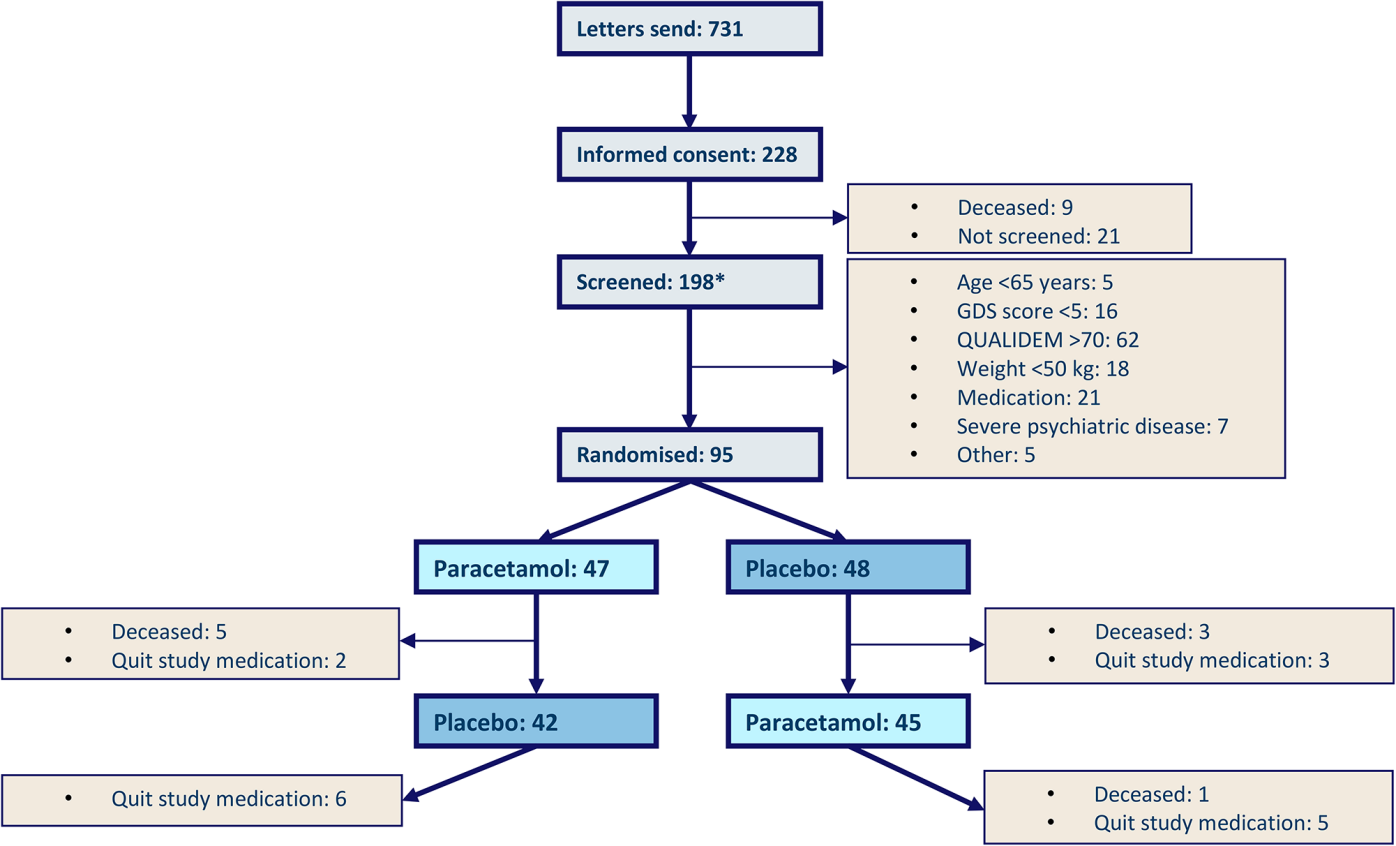
Flowchart of the Q-PID trial * Some overlap exists in the number of stated reasons for exclusion, because some persons met more than 1 exclusion criterium

A total of 95 persons with a mean age of 83.9 years (SD 7.6) were enrolled in the Q-PID study (Fig. 1). Data on care dependency and daily functioning were available for 94 persons (57.4% female) with a mean CDS total score of 37.8 (SD 12.9), and a mean Katz-15 total score of 11.9 (SD 2.4). The overall MOBID-2 pain score was 2.1 (SD 2.7), and 32.3% of the total group had clinically relevant pain (Table 1).

**Table 1:** Baseline characteristics of the total group, stratified by randomization group

	Paracetamol-placebo *N* = 46	Placebo-paracetamol *N* = 48	*P* value
Mean age (SD) in years	83.8 (7.5)	83.9 (7.7)	0.964^*^
Female (%)	26 (56.5)	28 (58.3)	0.859^**^
GDS score (%)			
5 6 7	2 (4.3) 34 (73.9) 10 (21.7)	6 (12.5) 32 (66.7) 10 (20.8)	0.157^**^ 0.443^**^ 0.915^**^
FCI (SD)	2.9 (2.0)	2.5 (2.1)	0.420^*^
CDS (SD)	39.7 (13.7)	36.0 (11.9)	0.171^*^
Katz-15 (SD)	11.5 (2.5)	12.3 (2.2)	0.100^*^
Pain (MOBID-2 ≥ 3) (%)	15 (33.3)	15 (31.3)	0.830^**^
MOBID-2 overall pain intensity (SD)	2.0 (2.4)	2.3 (3.0)	0.531^*^
Psychotropic use (%)	29 (60.4)	17 (37.0)	0.023^**^

^*^Unpaired t-test

^**^χ^2^ test

SD = standard deviation; GDS score = Global deterioration Scale (stage of dementia); FCI = Functional Comorbidity Index (0–18; higher score means more comorbidity); MOBID-2 = Mobilization-Observation-Behaviour-Intensity-Dementia-2 pain scale (0–10; ≥ 3 means clinically significant pain); CDS = Care Dependency Scale (15–75; higher score means less care dependency); Katz-15 (0 = 15; higher score means more help needed with tasks)

The characteristics of and measurements in the two randomization groups did not differ significantly at baseline, except regarding the number of persons who used psychotropics (antipsychotics, antidepressants, anxiolytics, hypnotics, and anti-dementia drugs); 37.0% in the paracetamol-placebo group vs. 60.4% in the placebo-paracetamol group (*p* = 0.023). Nine participants deceased during the study, resulting in 86 participants remaining at the end of the study periods (Fig. 1).

**Fig. 2:**
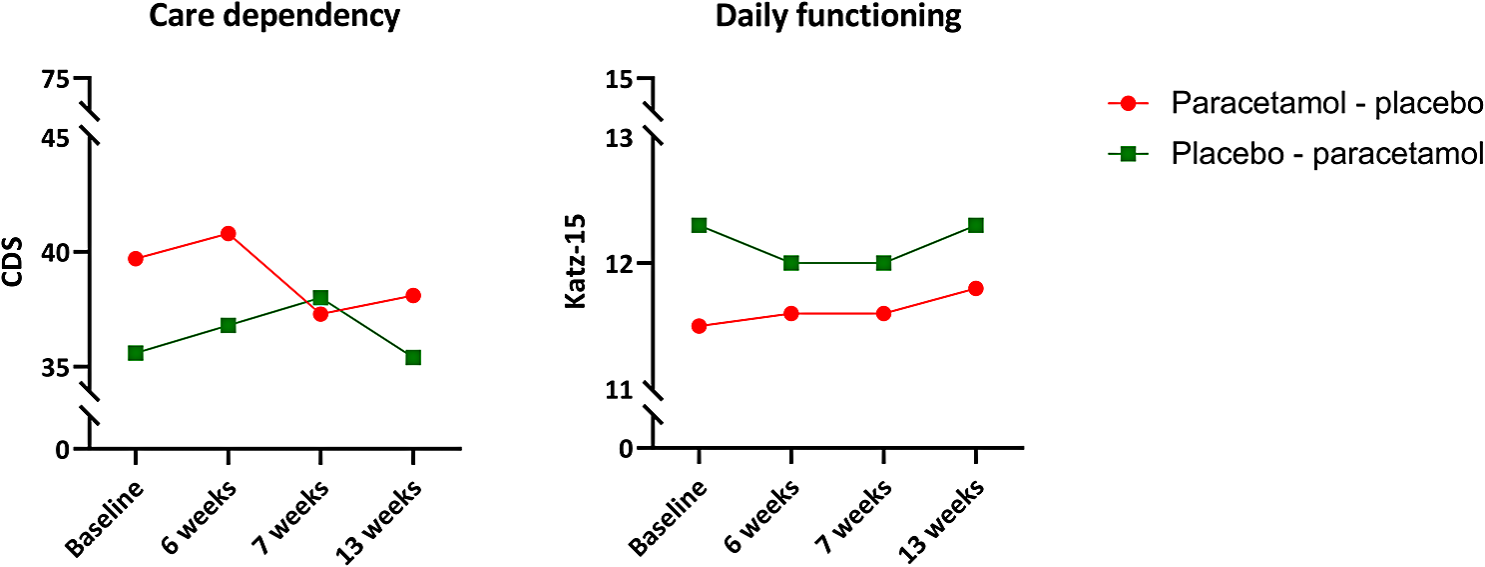
Mean CDS and Katz-15 total scores in the two treatment groups during the Q-PID study CDS: Care Dependency scale, range 15 (completely care dependent) to 75 (completely care independent) Katz-15: daily functioning, range 0 (completely independent functioning) to 15 (completely dependent functioning) Paracetamol - placebo: baseline − 6 weeks paracetamol, 7 weeks − 13 weeks placebo Placebo - paracetamol: baseline − 6 weeks placebo, 7 weeks − 13 weeks paracetamol

Repeated linear mixed models, adjusted for order and period effects, and psychotropic use, showed no difference in mean differences of care dependency (paracetamol − 1.0 [95% CI -2.4-0.3], placebo + 0.1 [-1.3-1.5]), and daily functioning (paracetamol + 0.2 [95% CI -0.2-0.6], placebo + 0.1 [-0.3-0.4]), in favour of either paracetamol or placebo (Table 2). Figure 2 shows the course of care dependency and daily functioning during the Q-PID study.

**Table 2:** Treatment effects of paracetamol and placebo on care dependency and daily functioning*N = 94 (baseline) *N* = 86 (end of study)

	Intervention	Mean difference	95% CI	*P* value
CDS^†^	Paracetamol	-1.0	-2.4–0.3	0.239
	Placebo	0.1	-1.3–1.5	
Katz-15^‡^	Paracetamol	0.2	-0.2–0.6	0.573
	Placebo	0.1	-0.3–0.4	

^*^ Repeated linear mixed models, adjusted for order and period effects, and use of psychotropics

^†^ Higher score means less care dependency

^‡^ Higher score means worse daily functioning

CI = Confidence interval; CDS = Care Dependency Scale (short version); Katz-15 = ADL and iADL scale 15 items

## Discussion

The aim of this study was to investigate the effect of paracetamol on care dependency and daily functioning. No statistically significant treatment effect of scheduled administration of paracetamol was found on care dependency and daily functioning in persons with advanced dementia with low QoL living in LTCF.

One explanation why we did not find an effect on care dependency and daily functioning may be the selection of patients. Participants were included in the Q-PID study based on having a low QoL and not using daily pain medication. The amount of pain was not a selection criterion, and was unknown prior to inclusion in the study. We found that 31.9% of the participants in the study had clinically relevant pain at baseline, and the mean MOBID-2 pain score of all 94 participants was 2.1 (SD 2.7), which is below the threshold of 3 (clinically relevant pain). This is lower than the numbers of persons with advanced dementia with pain found in other studies [1, 24, 25]. Consequently, the range of improvement of pain was already low for two-thirds of our study population. Other studies have demonstrated that pain may have a negative impact on care dependency and ADL functioning [1, 2]. As we found no significant improvement on pain [9], this might be a reason why care dependency and daily functioning did not improve either in the study. Moreover, prior to the Q-PID study, we hypothesized that when a person would feel better when paracetamol was taken, he/she may become less care dependent, and have a better QoL. However, no positive effect of paracetamol on QoL and wellbeing was found. With the progression of dementia, it is possible that care dependency and daily functioning may not be influenced easily, especially in persons that are not in pain. Finally, only small changes over time were seen in both intervention groups, especially regarding ADL functioning, and to a lesser extent also for care dependency. This was possibly based on natural variability in conjunction with the progression of the dementia, which may not be influenced by paracetamol (or placebo). These observed small changes over time of the Katz-15 (and CDS) may raise questions about the usefulness of these measures in clinical practice for persons with advanced dementia living in LTCF. The Katz-15 was designed to be used for research, and was validated for use in relatively healthy community dwelling older persons, the CDS to be used in nursing homes. Since the answers of the CDS are more defined with five options per item (‘completely independent’ to ‘completely dependent’), this measure may provide more leads as to which specific interventions are needed to improve daily care for these persons, compared to the ‘yes’ or ‘no’ answers regarding which items the person needs help with (Katz-15).

The most important strengths to mention are that the Q-PID study is one of the largest randomized double-blind placebo-controlled cross-over studies performed in LTCF among persons with advanced dementia, and that the number of drop-outs/withdrawals was within the prior estimated range. Also, a crossover design is an efficient design that causes less variance between measurements, and less confounding since the participants are their own controls. An important limitation was that a period effect was found, i.e., the total population had better QoL, and less neuropsychiatric symptoms in study period 1 compared to study period 2. This effect may have been caused by deterioration due to the disease, or by a Hawthorne effect for the first period (participation in a study in itself may lead to improvement). Furthermore, the compliance of the study medication in the second study period was lower than in the first study period, which was probably caused by a combination of type of administration (not in the same unit-dose packages as the other medications), and the workload of the nursing staff.

## Conclusions and implications

Compared to placebo, no effect of scheduled administration of paracetamol was found on care dependency and daily functioning in a group of 95 persons with advanced dementia with low QoL living in LTCF. It may be relevant for clinicians and nursing staff to find out more about the relationship between the different items of care dependency and existent pain, and which specific items of care dependency need special attention, to have points of reference to improve the care for, and thereby improve the QoL of, persons with advanced dementia. A multi-domain approach of professionals and informal caregivers, including non-pharmacological interventions, is essential to reach this goal.

## Data Availability

The datasets used and/or analysed during the current study are available from the corresponding author on reasonable request.
